# Dietary MicroRNA Database (DMD): An Archive Database and Analytic Tool for Food-Borne microRNAs

**DOI:** 10.1371/journal.pone.0128089

**Published:** 2015-06-01

**Authors:** Kevin Chiang, Jiang Shu, Janos Zempleni, Juan Cui

**Affiliations:** 1 Department of Computer Science and Engineering, University of Nebraska-Lincoln, Lincoln, NE, United States of America; 2 Department of Nutrition and Health Sciences, University of Nebraska-Lincoln, Lincoln, NE, United States of America; Shenzhen Institutes of Advanced Technology, CHINA

## Abstract

With the advent of high throughput technology, a huge amount of microRNA information has been added to the growing body of knowledge for non-coding RNAs. Here we present the Dietary MicroRNA Databases (DMD), the first repository for archiving and analyzing the published and novel microRNAs discovered in dietary resources. Currently there are fifteen types of dietary species, such as apple, grape, cow milk, and cow fat, included in the database originating from 9 plant and 5 animal species. Annotation for each entry, a mature microRNA indexed as DM0000*, covers information of the mature sequences, genome locations, hairpin structures of parental pre-microRNAs, cross-species sequence comparison, disease relevance, and the experimentally validated gene targets. Furthermore, a few functional analyses including target prediction, pathway enrichment and gene network construction have been integrated into the system, which enable users to generate functional insights through viewing the functional pathways and building protein-protein interaction networks associated with each microRNA. Another unique feature of DMD is that it provides a feature generator where a total of 411 descriptive attributes can be calculated for any given microRNAs based on their sequences and structures. DMD would be particularly useful for research groups studying microRNA regulation from a nutrition point of view. The database can be accessed at http://sbbi.unl.edu/dmd/.

## Introduction

Empowered by revolutionary sequencing technology, microRNAs have been extensively discovered in various dietary resources including plants (e.g. rice and tomato) and animals (e.g. milk and meats). Given the broad implications of microRNA in health and disease [1–8], research enthusiasm for functional impacts of exogenous food microRNA in human cellular phenotypes has soared, which warrants the efforts to build related bioinformatics tools and databases. The Dietary MicroRNA Database (DMD) represents the first repository in this domain for archiving and distributing the published food-borne microRNAs in literatures and public databases.

There are several public databases focused on microRNA identification and targets prediction that archive validated microRNAs with sequence, structure and interaction information. For example, miRBase (http://www.mirbase.org) records 64,473 microRNAs from 223 species [9] and MiRecords [10] hosts 2,705 records of interactions between 644 microRNAs and 1,901 target genes in 9 animal species. Databases such as TargetScan [11], Miranda [12] and MirTarBase [13] provide information of the validated gene targets as well as the computationally predicted targets. For example, 60% of human genes are regulated by microRNAs, participating in many major cellular processes such as cell growth, differentiation and apoptosis [14, 15]. In addition, microRNA expression data, although limited, are archived in public databases such as GEO databases [16] and TCGA [17]. However, none of the aforementioned databases cover dietary information that may represent new horizon in microRNA research. For example, miRBase has reported 808 microRNAs in bovine, whereas only 243 of them have been found in cow milk [18] and 213 in the fat of cow beef [19]. Likewise, human breast milk only contains 434 microRNAs, out of the total of 2,588 microRNAs in human [20]. We envision such diet-specific cohorts would be important for nutritionists and general biologists to investigate microRNA dietary intake and analyze subsequent regulations in human health and diseases. Expelling evidences sustaining our hypothesis include the following: it has been recently discovered that human can absorb certain exosomal microRNAs from cow’s milk, e.g., miR-29b and 200c, and that endogenous microRNA synthesis does not compensate for dietary deficiency [21]; the biogenesis and function of such exogenous miRNAs are evidently health related [21–24]. However, while the evidence in support of bioavailability of milk miRNAs is unambiguous, a recent report that mammals can also absorb plant miRNAs (e.g. miR-168a) from rice [25] was met with widespread skepticism [26–29]. Based on these evidences, challenging questions may be raised regarding how humans pick up microRNAs from diet and what are the broader roles played by such exogenous microRNAs in human disease processes.

In order to facilitate more advanced research related to dietary microRNAs, DMD was developed as the first repository for archiving and analyzing the published microRNAs discovered in dietary plants and animals, such as cow milk, breast milk, grape, beef, pork, apple, banana and etc. For each reported microRNA, various types of information have been covered, including sequences, genome locations, hairpin structures of parental pre-microRNAs, disease relevance, and experimentally validated gene targets. We also integrate an analytical pipeline into this platform that includes cross-species sequence comparison, target prediction, gene enrichment analysis and microRNA-mediated gene network construction, which we will introduce in the following sections.

Compared to other microRNA-related databases, DMD also has a few unique features. For example, a feature generation tool allows users to calculate a comprehensive set of molecular discriminators based on the sequences and structures of any microRNA entry in the database or uploaded on their own. These discriminators have been considered as important features for microRNA identification and microRNA-mRNA interaction prediction and have been employed by many current tools in addition to the use of complementary seed sequences as major motifs in animal and plant species [11, 30–34]. Based on the targets, one can extract the functional pathways information and infer the functional impacts of the microRNAs through their gene regulation [35, 36]. In the later section, we will use a case study to demonstrate the usefulness of this database.

## Materials and Methods

### Database Construction and Access

**Fig 1:**
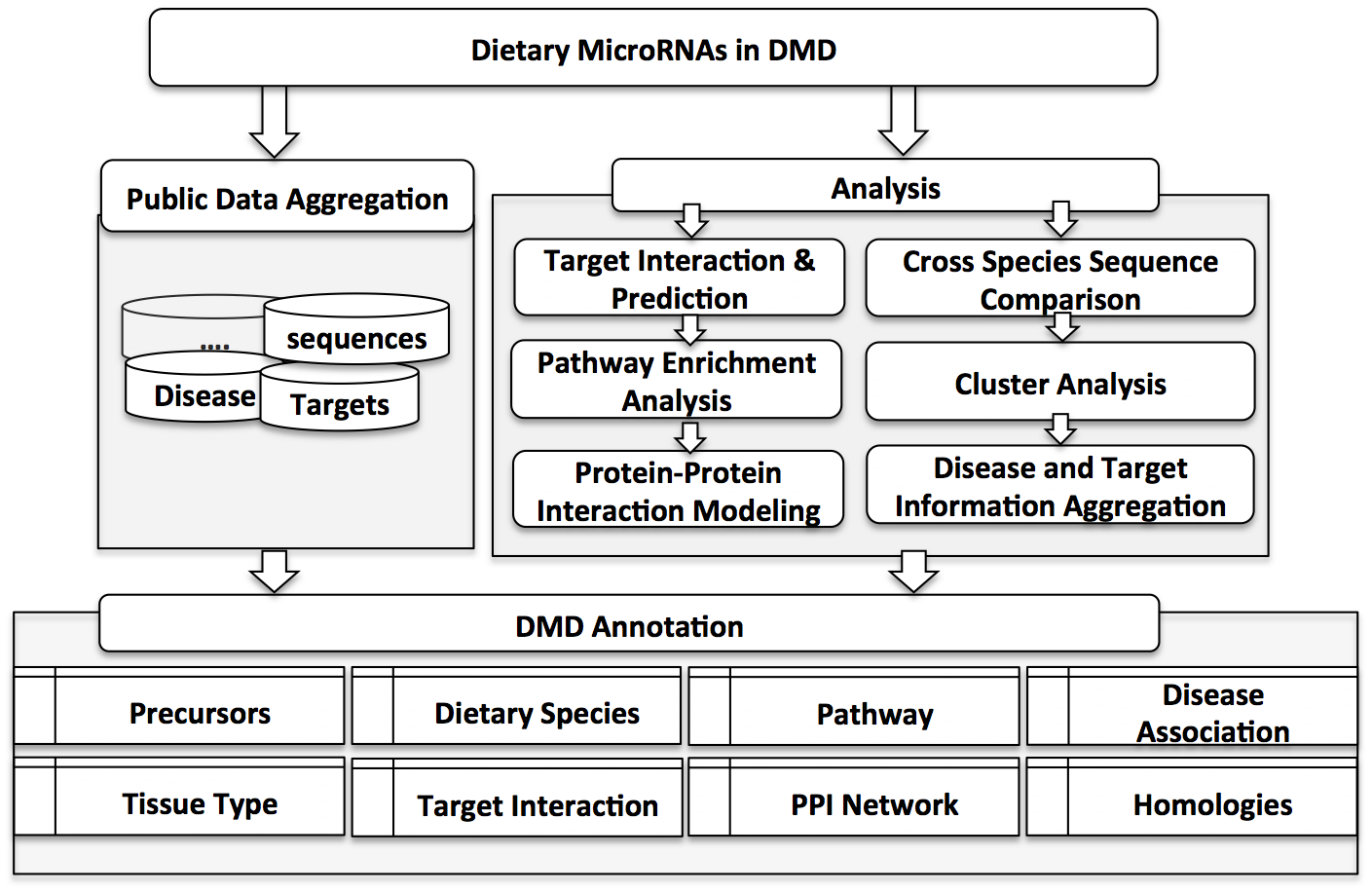
DMD construction workflow and the outline of data content.

Fig 1 shows the workflow of the data collection and analysis with DMD. Through literature and database search, we compiled and reported microRNAs from 15 types of dietary species. For each entry, the basic annotation page includes ten types of information including mature sequences, genome coordinates, pre-microRNA sequence, hairpin structure, cross-species sequence comparison, disease relevance, and the experimentally validated and predicted gene targets. For entries from public databases, e.g. miRBase, we have provided links to the external annotation pages.

The DMD was created using a MySQL database, consisting of 25 tables (S1 Fig). The outline (Fig 1) shows that the database content can be categorized into three areas, namely basics, annotation and analysis. First, many external databases are integrated within DMD to allow for quick viewing and annotation of microRNAs. Second, there are the prediction tools, which allow users to quickly view homologous microRNAs via the clustering analysis by CD-HIT [37] and predict gene targets in their own species and in human. Finally, there is an intensive process to annotate microRNAs into dietary species and tissues.

The S1 Fig shows the database design, table relationships and indexing patterns among these tables. All information, including from external databases, to prediction tools and annotation, are heavily connected, as can be seen from the schema. This allows for the information within the database to be shared easily and quickly.

In addition to the MySQL databases, the graph database Neo4j (http://neo4j.org) was used to model protein-protein interaction. The graph database consists of two different types of nodes, a microRNA and a protein node, and two types of edges, a protein-protein interaction undirected edge, and a microRNA to protein regulation directed edge.

The information of DMD can be freely accessed from http://sbbi.unl.edu/dmd/. Data submission and download can be accessed through a secure user login system.

### Cross-species Sequence Comparison

In order to assess the sequence conservation of each microRNA during evolution, we conducted sequence alignment and comparison using CD-HIT [37] where the microRNA with similar sequences can be grouped into the same clusters. In this analysis, each cluster represents a collection of microRNAs that share identical or highly similar sequences (with identity higher than 95% of the sequence length), which could originate from various species, e.g. homologous microRNAs. Within the cluster, the user will be able to view the microRNA name, sequence alignment, associated gene targets and diseases, along with the option of viewing information among diet-only or all species.

### Querying the Database (Browsing and Searching)

Users are able to browse microRNAs by species using the browse page. Accessing each species will output a whole list of microRNAs specifically discovered in that dietary species. For example, microRNAs under “cow milk” and “cow fat” are subsets of all known microRNAs from bovine organism. In addition to being able to browse by species, there are three methods of searching the datasets, by “ID” (DMD index number, e.g. DM00001), “Name” (microRNA name, e.g. bta-miR-29b or part of the name, e.g. 29b) or “Sequence”(either mature or pre-microRNA sequences, e.g. ugagguaguagguuguauaguu). Again, the search can be constrained within mature microRNAs or precursors according to the user defined criteria. All outputs from the search are organized initially by their unique DMD identifiers, but the results may be re-sorted by microRNA name, or sequence.

### Experimental and Predicted Targets

Experimental targets information was extracted from miRTarBase [38], which contains 18 species and 51,460 miRNA-target interactions. A few of computational tools were included for target prediction, especially for species without validated target information, including MirTarget [39], targetScan [11] and psRNAtarget [40]. Please note prediction could be specifically designed for certain organism, e.g. human specific or plant specific. All microRNA sequences will be also subject to a target prediction against human genome no matter whether predictions on their own genomes are available or not.

### Functional Analysis Based on MicroRNA Targets

According to the predicted and experimental targets for each microRNA entry, users can choose to run a pathway enrichment analysis on selected targets. 1,955 pathways from KEGG [41] are included. Modified p-value was calculated for each relevant pathway based on Fisher’s exact test on queried targets against the whole genome. In addition to the pathway enrichment analysis, protein-protein interaction (PPI) network [42] was employed to visualize the microRNA-mRNA regulation network.

### Feature Construction

As molecular properties of microRNA sequences and structures are key for target identification, we have developed a *feature* page to allow users to calculate for any given microRNA sequences a list of features categorized into two classes: sequence-based features and secondary structure features. Particularly, for each mature miRNA, features were generated on both mature sequences and the corresponding pre-miRNA sequences, such as existence of palindromic sequences, sequence length and the composition of monomers and dimers. Such features have been shown to be discriminants when used for machine learning [18, 31, 32, 43–46].

Secondary structure features were calculated based on the pre-miRNA sequences. For example, RNAfold [47] was employed to predict secondary structure and calculate Minimum Free Energy (MFE) [48]. Based on the predicted structure of pre-miRNA, 32 triplet features and 11 base-paired features were calculated, such as A ((((the frequency of 3 paired nucleotides leading with A) and %pairGC (percentage of the paired G-C bases). Additionally, RNAshape was used to map secondary structures to tree-like domain of shapes, retaining adjacency and nesting of structural features, but disregarding helix length [49]. STOAT, packaged in the NOBAI web server, was utilized to compute Shannon Entropy (Q) and Frobenius Norm (F) [50]. See Table 1 for a complete list of features.

**Table 1:** List of features available for generation.

Category	Feature Details	Feature Dimensions	Reference
**Primary Sequence**	Single Nucleotide Frequency	4 x 3^1^	[31, 46]
	Pairwise Nucleotide Frequency	16 x 3^1^	[31, 32, 43, 46]
	Triplet Nucleotide Frequency	64 x 3^1^	[31, 43, 46]
	Quadruplet Nucleotide Frequency	256 x 3^1^	[46]
	A + U Frequency	1 x 3^1^	[43, 44]
	G + C Frequency	1 x 3^1^	[31, 32, 43, 44, 46]
	G + U Frequency	1 x 3 ^1^	[43, 44, 46]
	Number of Palindromes in Sequence	1 x 3^1^	[45]
	Length	1 x 3^1^	[31]
	Pairs of A-U in Premature microRNA	1	[43]
	Pairs of G-C in Premature microRNA	1	[43, 44]
	Pairs of G-U in Premature microRNA	1	[43]
**Secondary Structure**	Nucleotide to RNAfold^2^ triplet match. (A(((, C(.(, G(… etc…)	32	[44, 46]
	Minimum Free Energy, Normalized Minimum Free Energy, Frequency of Minimum Free Energy Structures	3	[31, 32, 43, 44]
	Ensemble Free Energy, Normalized Ensemble Free Energy	2	[32, 44]
	Stem Statistics (Stems, Average Stem Length, Maximum Stem Length, Stem containing AU, Stem containing GC, Stem containing GU)	6	[32, 43, 44]
	Minimum Free Energy Statistics (mfe/G+C frequency, mfe/stems, mfe/unpaired nucleotides, mfe/paired nucleotides, difference in mfe and efe, and ensemble diversity).	6	[32, 43, 44]
	Percentage of sequence composing of pairs.	1	[46]
	Frequency of Nucleotides that occur outside of UA, GU, GC pairs.	4	[46]
	Predicted shape type probability base on RNAshapes^3^.	5	[51]
	STOAT^4^ statistics (Shannon Entropy, Frobenius Norm, Base-pairing propensity, and mean stem length)	4	[32]

^1^These features may be calculated for the premature sequence, mature sequence, and seed region sequence.

^2^RNAfold is an external tool that is run with the—p option to generate the partition function and base pairing probability.

^3^RNAshapes is an external tool that is run with the—t option to specify 5 different shape types.

^4^STOAT is an external tool that is run with the—x 31 option to signify 31 character states and the—v option to display a verbose option that is easier to parse.

## Results and Discussion

As a dietary microRNA database, DMD acts as the first repository archiving microRNA sequence and annotation that are related to any dietary species. Currently there are 15 dietary species have been curated in DMD, including five animal species (human, chicken, cow, pig and salmon) and nine plant species (soybean, tomato, corn, apple, orange, banana, grape, rice, and wheat). Please note that dietary species might originate from the same biological organism. For example, the bovine microRNAs are organized under different dietary groups, including cow milk and cow fat. Table 2 shows the statistics of different types of information archived for each species.

**Table 2:** Statistics of microRNAs and species in DMD.

*Types*	*Species*	*Mature [9]*	*Precursor [9]*	*Exp*. *Target*	*Pred. Target [38, 40]*
		*#*. *of dietary miRNAs/#*. *of known miRNAs in the organism*		*[38]*	
***Animal***	*Human Breastmilk*	*434/ 2588*	*402 / 1881*	*9995*	*17*,*416*
	*Cow Milk*	*243 / 793*	*245 / 808*	*3*	*16*,*451*
	*Cow Fat*	*205 / 793*	*229 / 808*	*5*	*16*,*269*
	*Atlantic Salmon*	*498 / 498*	*371 / 371*	*-*	*17*,*319*
	*Chicken*	*994 / 994*	*740 / 740*	*19*	*18*,*075*
	*Pig*	*411 / 411*	*382 / 382*	*-*	*17*,*406*
***Plant***	*Apple*	*203 / 207*	*202 / 206*	*-*	*13*,*098*
	*Banana*	*360 / 360*	*180 / 180*	*-*	*16*,*537*
	*Corn*	*309 / 321*	*166 / 172*	*-*	*16*,*456*
	*Grape*	*108 / 186*	*157 / 163*	*-*	*12*,*236*
	*Orange*	*047 / 064*	*057 / 060*	*-*	*12*,*655*
	*Rice*	*634 / 713*	*526 / 592*	*-*	*21*,*355*
	*Soybean*	*620 / 639*	*554 / 573*	*-*	*20*,*261*
	*Tomato*	*040 / 110*	*068 / 77*	*-*	*10*,*827*
	*Wheat*	*111 / 119*	*108 / 116*	*-*	*15*,*327*

### Database Content

The information stored in DMD is categorized into *Sequences* and *Annotation*.

Sequences: Currently, there are 11,569 microRNA entries in DMD, including 5,217 unique mature sequences and 5,865 unique pre-microRNA sequences. Duplicates contribute to the total microRNA, which is due to a microRNA being present in multiple dietary species. DMD follows the same naming standard for each entry, e.g. microRNA gene names have the form hsa-miR-200, consistent with other databases, e.g. miRBase. The prefix signifies the organism, in this case Homo Sapiens. Each entry in the database, indexed as DM0000*, represents the mature sequence, with the information on the genomic location and hairpin sequence of the parental pre-microRNA, indexed as DP0000*, which will have the corresponding miRBase index if entries from both database are the same. Homologous microRNA loci in different species are assigned the same number. Paralogous microRNAs are assigned names with lettered and numbered suffixes, depending on whether the derived mature microRNA is identical in sequence, or contains sequence differences. The derived mature microRNAs were previously assigned names of the form dme-miR-100 and dme-miR-100*, for the guide and passenger strand, respectively while hsa-miR-100-5p and hsa-miR-100-3p were assigned for sequences derived from the 5’ and 3’ arms of the hsa-miR-100 hairpin precursor.

*Annotation*: In addition to the general information such as sequence and structure, DMD has also generated features for each of the microRNAs. The *feature* page provides users with 411 molecular attributes that can be calculated based on the microRNA sequences and structures. Table 1 lists all the features included in this study, and also presents a non-exhaustive list of studies that have reported use of a particular feature. Other annotation information covers targets, sequence comparison against other species, and pathway and interaction network analysis based on the either experimentally validated targets or computational predicted targets. Specifically, the pathway information was compiled from KEGG. PPI information has also been used to visualize the interaction network based on the given targets. Furthermore, microRNA disease information was extracted from mirCancer and obesity, the Human microRNA Disease Database [52], PhenomiR [53], and PubMed literature search.

The *browse* page allows users to access microRNAs under each dietary species while the database metadata can be downloaded as plain text. For each microRNA sequence entry, there are links to other databases providing the primary references that describe its discovery, links to the evidence supporting the microRNA annotation, genomic coordinates and links to databases of predicted and validated microRNA target sites. Entries can be searched either by sequence or keyword. Note the name cannot be used as a substitute for rigorous sequence analysis.

### Unique Analytical Workflow

DMD offers not only the basic access to sequence and annotation data stored in the database but also a few analytical tools integrated as the web services. For examples, the feature page allows users to calculate for any given microRNA a list of 411 features based on the sequences and structures; the cross-species sequence comparison was integrated on each entry page. Other analyses are focused on the functional inference through microRNA target identification.

#### Target prediction

On each annotation page for a given microRNA, we have provided targets from both experimental studies and computational prediction. A total of 91,032 experimental targets have been included in this database while computational predictions are mainly through MirTarget and psRNAtarget. Since the target prediction could be organism specific, we separated the cases for plant and animal and provide for each microRNA the potential targets in human.

#### Functional inference and Network Analysis

Pathway enrichment analysis and gene network construction have been implemented for users who are interested in functional analysis. Protein-protein interaction visualization network can be seen from a microRNA entry’s predicted and experimental targets. First, selecting from the list of targets will display a new list, which shows the interactions between two targets with up to three intermediates. Opting to show the interaction will open a new window, which visualizes the interaction between the two targets. Included in the network are the two selected targets, all its shared gene intermediates, and all the microRNAs that regulate the two targets. We will illustrate in the following section of these analyses.

In addition to the analysis, the password-protected *upload* page allows registered users to deposit newly identified evidences of microRNA occurrences in food. Novel microRNAs can be submitted after an article describing their identification is accepted for publication in a peer-reviewed journal. Since this field needs fast gain of knowledge, we also allow users to upload their raw data prior to the publication, for instance, a single microRNA detected based on PCR or a raw microRNA array or sequencing data that might under review or in-house use. We will take these data in and analyze internally using the broadly used bioinformatics standards.

### An Application Example to Reconstruct microRNA-29b Regulation Network

**Fig 2:**
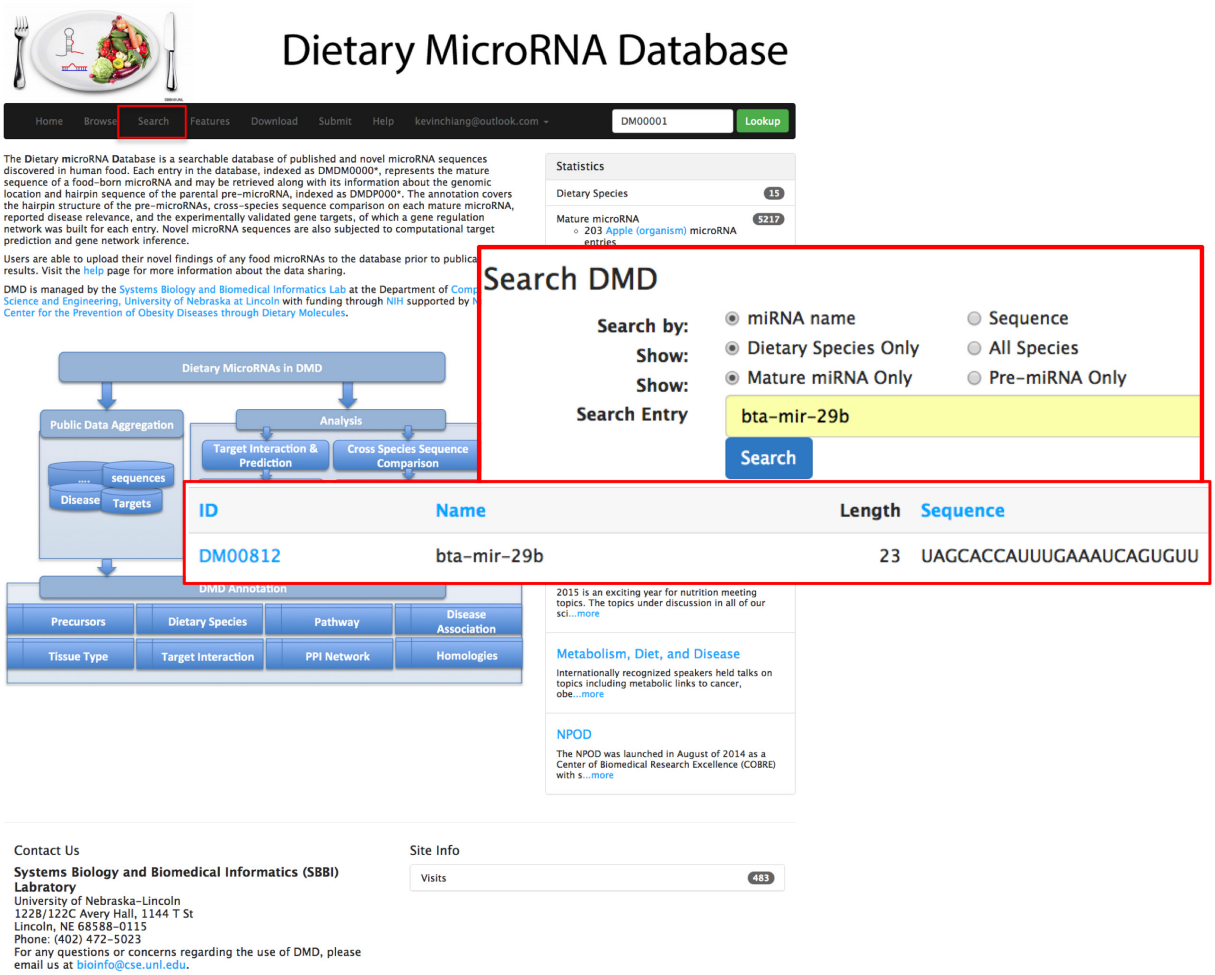
Illustration of searching "bta-mir-29b" and the search results.

Bta-miR-29b, a cow-milk microRNA that has been discovered in individuals involved in a milk feeding study [21], is used for illustration of searching and analyzing a specific microRNA in DMD. Fig 2 shows the returned entry when user searching by microRNA name on the search page.

**Fig 3:**
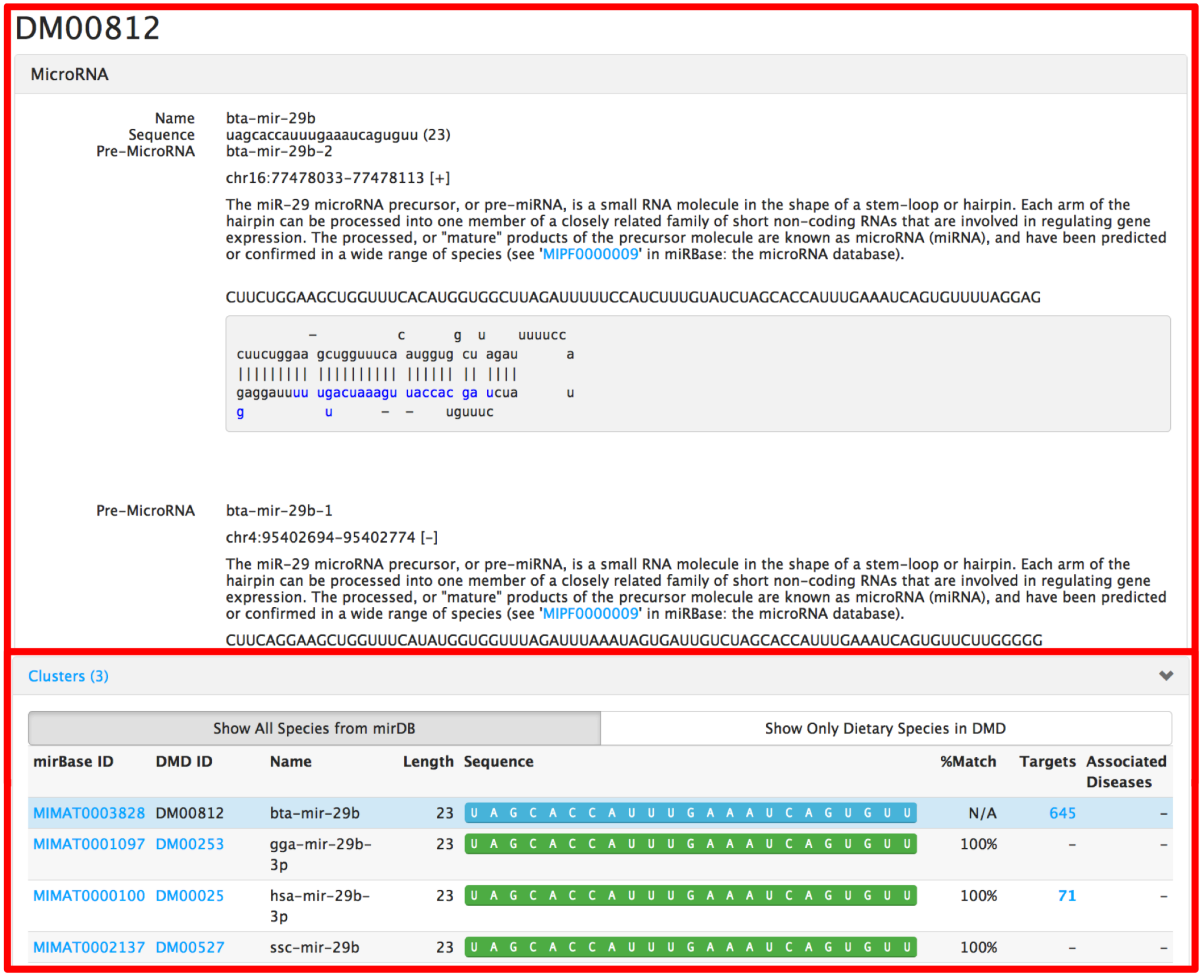
The DMD entry for 'bta-mir-29b,' which contains sequence information, precursor annotation, and homologous sequences and their associated targets and diseases.

**Fig 4:**
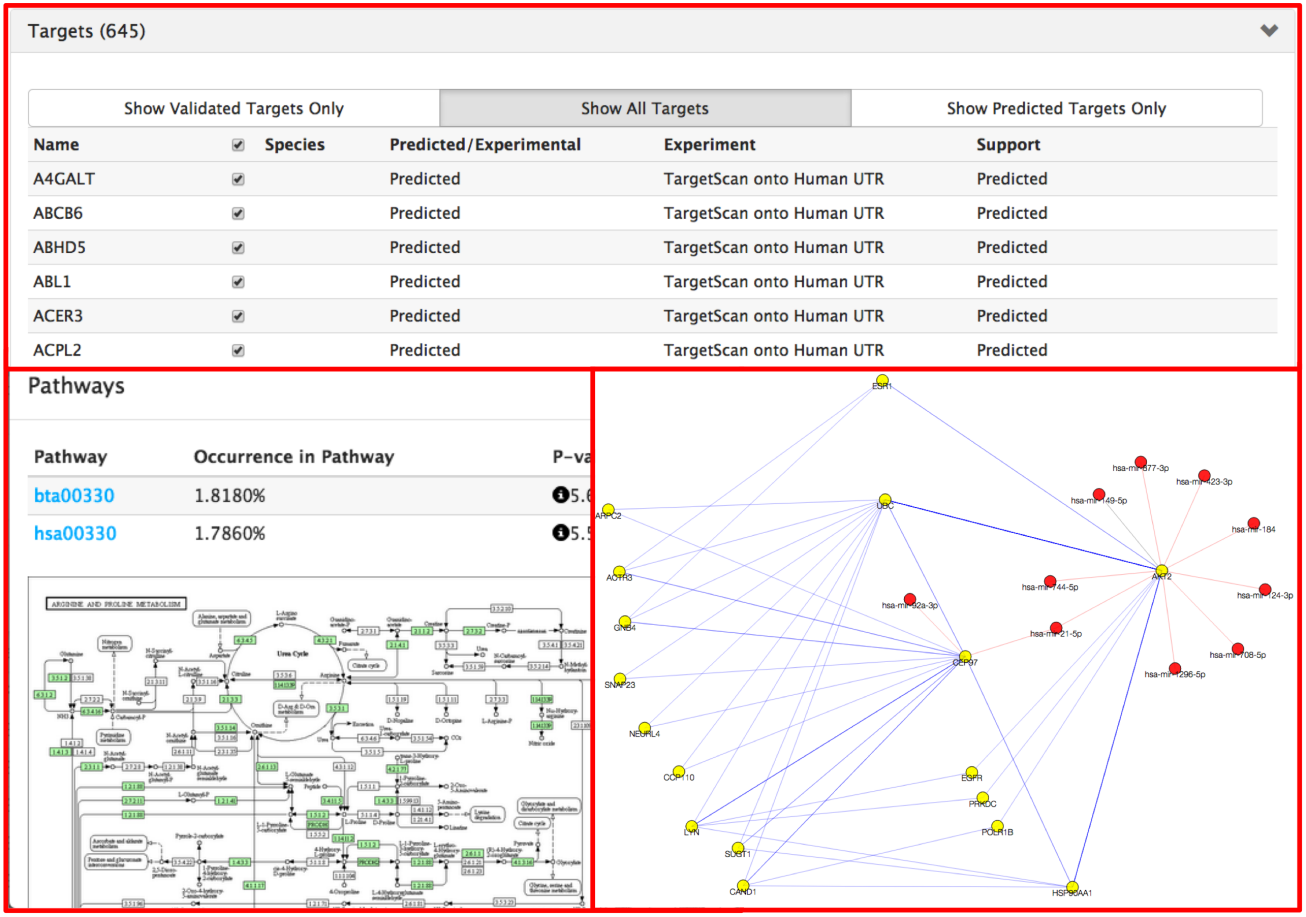
Clockwise from top: "bta-mir-29b” shows 645 targets; the protein-protein network visualization; and the gene enrichment analysis and pathway information.

Selecting the ID DM00812 leads to the annotation page shown in Fig 3 and Fig 4. In the top panel of Fig 3, it gives the basic sequence information along with its precursor sequence, structure, and coordinates. The panel labeled “Clusters” shows a list of homologs that have at least 95% sequence similarity in other species. In this case it shows the mir-29b homologs for chicken, human, and pig. This panel also shows the number of targets for each homolog as well as associated diseases, if available. The top panel labeled “Targets” in Fig 4 shows experimentally validated and computationally predicted gene targets. Users have the option to run gene set enrichment analysis on the selected targets as well as visualize the protein-protein network.

**Fig 5:**
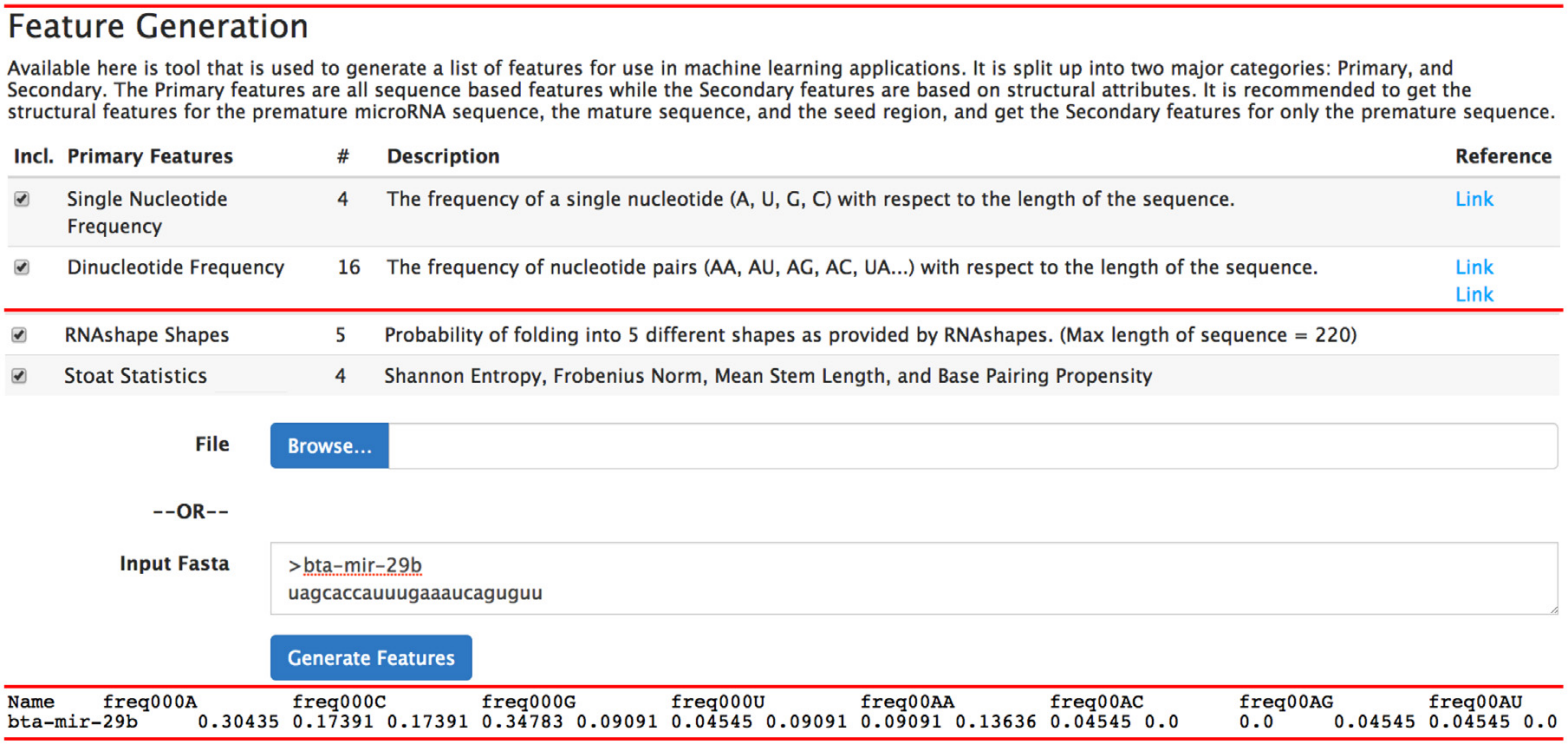
The feature generation page showing the entry of “bta-mir-29b” in fasta format and its output in a tab separated values format.

To use bta-mir-29b in a data mining capacity, users may then copy the microRNA sequence and navigate to the feature page and calculate up to 411 features (Fig 5). Users may also submit a fasta file to generate features. Clicking on “Generate Features” will begin the process of computationally generating the features listed in Table 2. Once complete, a page containing tab-separated values is presented to the user to copy and paste. The first line contains a header file describing the features generated.

## Concluding Remarks

Here, we introduce the Dietary MicroRNA Database (DMD) that distributes all published microRNA sequence from dietary resources, for browsing and searching by sequence and keywords, through a web interface (http://sbbi.unl.edu/dmd/). In addition, we have provided sequence comparison, target prediction and feature generation functionality on the websites.

## Future Development

We will continue the literature search for newly published data in other dietary species to update the database. We also plan to integrate the real-time analytical pipeline so users can submit their sequences and evaluate the results from their side. Another plan for this database is to let user upload paired microRNA and gene expression profiles where both mRNA expression changes and microRNA expression alterations under the same conditions can be captured, so that one can employ dynamic modeling [54] to infer the conditional-dependent dynamic regulation network.

## Supporting Information

S1 Fig DMD Database Schema. (TIFF)
