# Supplementary figures and images for: Dietary MicroRNA Database (DMD): An Archive Database and Analytic Tool for Food-Borne microRNAs
Source: PLoS One. 2015 Jun 1;10(6):e0128089. doi: 10.1371/journal.pone.0128089 (PMC4451068; doi:10.1371/journal.pone.0128089)

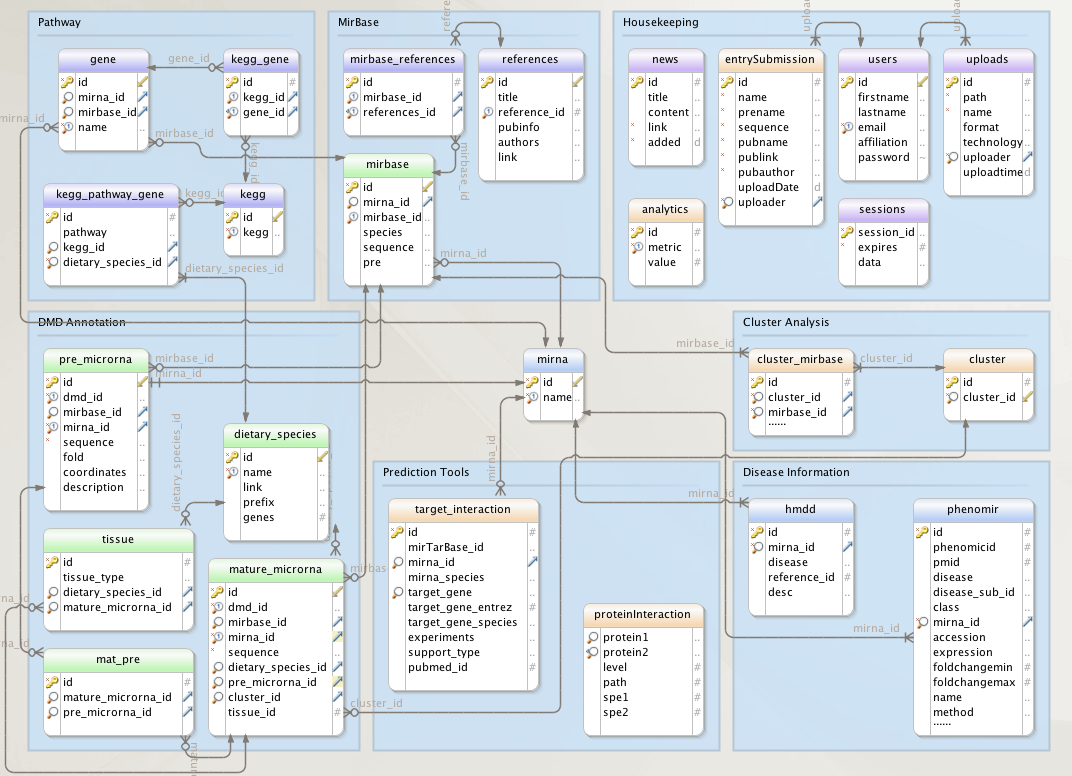

Supplement: S1 Fig — (TIFF) [file pone.0128089.s001.tiff]
